# Energy-Efficient EEG-Based Scheme for Autism Spectrum Disorder Detection Using Wearable Sensors

**DOI:** 10.3390/s23042228

**Published:** 2023-02-16

**Authors:** Sarah Alhassan, Adel Soudani, Manan Almusallam

**Affiliations:** 1 Department of Computer Science, College of Computer and Information Science, King Saud University, Riyadh 11362, Saudi Arabia; 2 Department of Computer Science, College of Computer and Information Science, Imam Mohammad Ibn Saud Islamic University, Riyadh 11564, Saudi Arabia

**Keywords:** Autism Spectrum Disorder detection, wearable sensors, EEG signal, on-node feature extraction and classification, embedded machine learning

## Abstract

The deployment of wearable wireless systems that collect physiological indicators to aid in diagnosing neurological disorders represents a potential solution for the new generation of e-health systems. Electroencephalography (EEG), a recording of the brain’s electrical activity, is a promising physiological test for the diagnosis of autism spectrum disorders. It can identify the abnormalities of the neural system that are associated with autism spectrum disorders. However, streaming EEG samples remotely for classification can reduce the wireless sensor’s lifespan and creates doubt regarding the application’s feasibility. Therefore, decreasing data transmission may conserve sensor energy and extend the lifespan of wireless sensor networks. This paper suggests the development of a sensor-based scheme for early age autism detection. The proposed scheme implements an energy-efficient method for signal transformation allowing relevant feature extraction for accurate classification using machine learning algorithms. The experimental results indicate an accuracy of 96%, a sensitivity of 100%, and around 95% of F1 score for all used machine learning models. The results also show that our scheme energy consumption is 97% lower than streaming the raw EEG samples.

## 1. Introduction

Autism Spectrum Disorder (ASD) is among the most common childhood neurodevelopmental disorders (approximately 1 in 44 children) [1,2]. According to the American Psychological Association [3], ASD subjects often have restricted and repetitive activity patterns that characterize social, communication, and interaction difficulties. Early diagnosis of ASD can improve the quality of life for autistic children and their families [4] and significantly reduce the severity of later effects [5]. The situation is further complicated as the eligibility of many children for early intervention therapy lapses as they reach school age [6].

Current ASD diagnosis is based on subjective behavioural assessment derived from parent interviews and observations [7]. For an accurate diagnosis, an exhaustive analysis of the child’s skills is necessary, which might take months or even years, delaying the starting of accommodation therapy [8]. Furthermore, behaviour-based diagnosis is challenged by the fact that ASD symptoms may overlap with other neurodevelopmental disorders, especially in mild ASD cases [9].

An open research target is the investigation of new autism biomarkers to be used as accurate diagnostic tools that provide early ASD detection and informed clinicians’ decisions [10]. There is a need for a scalable ASD biomarker that can be used during standard check-ups [7,11]. This biomarker should be simple, affordable, behaviour-independent, and suitable for routine examinations [8].

Several neuroimaging and neurophysiological methods have been deployed to study the association between brain functionality and autism behaviour [12] to remove the bias of the current subjective behaviour-dependent diagnosis process. Among these methods, electroencephalography (EEG), a measurement of potentials through electrodes placed on the scalp that reflect the brain’s electrical activity [13,14], can serve as an investigational tool for characterizing neurodevelopmental disorders [15] and, in particular, for ASD diagnosis [5,9,16]. In fact, EEG-based early brain development evaluation can indicate ASD even before the onset of behavioural symptoms [7]. Even in the absence of ASD-related behaviour, the EEG signal contains temporal, frequency, and phase information that can aid in recognizing abnormal neural activity [17]. The diagnostic potential of EEG signals should, therefore, be capitalized in the development of assistive automatic EEG-based schemes for ASD detection.

Several studies discussed in [9] demonstrated evidence of EEG-based ASD detection. The extracted features from prior investigations are categorized into three primary groups [9]: information dynamics [7], functional connectivity [18,19,20], and spectral power [5,21,22].

**Figure 1:**
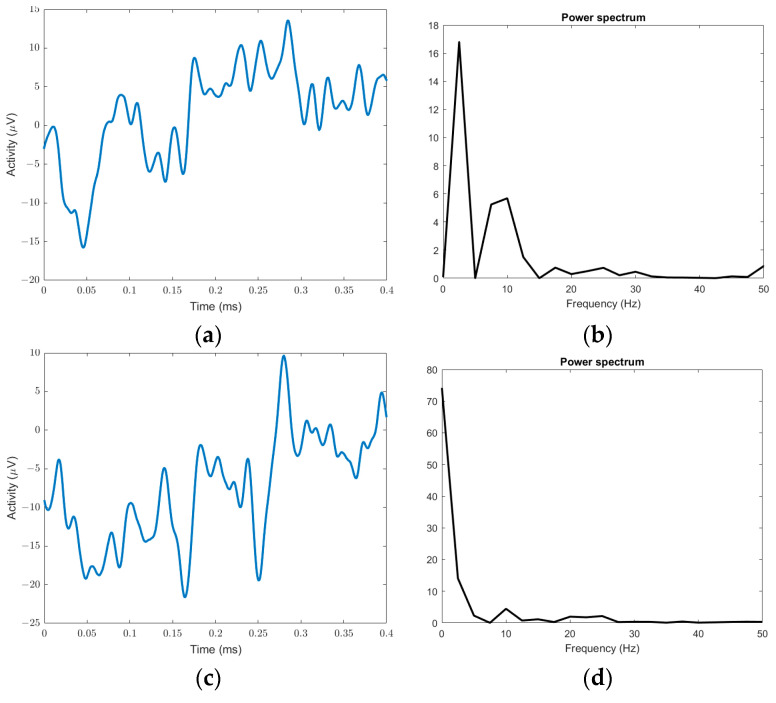

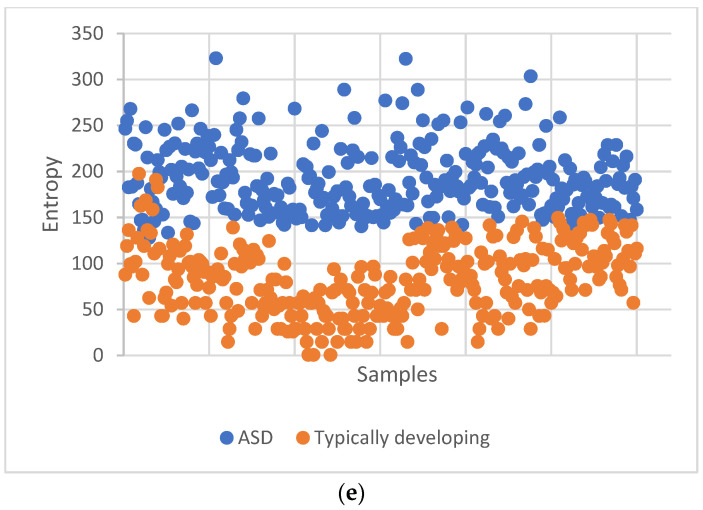
Sample EEG signals, their power spectrum, and the distribution of entropy dynamics between ASD and typically developing subjects. (**a**) ASD EEG sample, (**b**) power spectrum of ASD EEG sample, (**c**) typically developing EEG sample, (**d**) power spectrum of typically developing EEG sample, (**e**) distribution of entropy dynamics.

To confirm this statement, we illustrate in Figure 1 the distribution of power spectrum and entropy dynamics features extracted from EEG signals recorded for ASD cases and typically developing groups. Due to nonlinearity, dynamics, and complex characteristics, the EEG signals are difficult to observe and interpret visually [23]. However, the use of power spectrum and entropy dynamic features (Figure 1a–e) shows different distributions between ASD and typically developing subjects in brain function, which attests the feasibility of using EEG-based features for ASD detection.

Previous research contributions studied the analysis of EEG signal for ASD detection with different approaches and based on a variable set of features. Information dynamics features entail nonlinear approaches to find group differences, such as entropy features [9]. Bosl et al. [7] extracted nonlinear measures and claimed it was the first study to apply these measures to developmental neurobiology.

Spectral analysis is considered the most prevalent EEG approach [9]. It was used by Gabard-Durnam et al. [5]. In their approach, the EEG signal was decomposed using FFT, and then the summed power was calculated across all frequencies. Individual alpha peak frequency (iAPF) and individual alpha absolute power (iABP) features were extracted by Zhao et al. [21]. Few studies utilized machine learning in the classification. Bosl et al. [7] utilized the support vector machine (SVM) to produce classification results exceeding 95% in terms of specificity, sensitivity, and positive predictive value. In addition, Zhao et al. [21] employed the support vector machine to achieve 92% accuracy. Gabard-Durnam et al. [5] used logistic regression and reached 90% sensitivity.

Nevertheless, prior studies on EEG-based autism detection relied on wired sensors and did not address the challenges of EEG recording for autistic children. Extensive wiring and lengthy recording sessions often restrict the use of wired EEG sensors to laboratory settings [24,25]. In addition, a lengthy EEG preparation procedure is difficult and unpleasant for young children, which reduces the time available for practical testing [26].

On the other hand, wearable EEG sensors [24] offer inexpensive, non-invasive, and portable real-time monitoring of human brain activity. These sensors are key components in Internet of Medical Things (IoMT) [27] that emerged as the next-generation bioanalytical tools designed to improve the functionality and decision-making ability of healthcare applications. In depth, using wearable EEG sensors reduces the setup burden for EEG specialists and facilitates testing in children’s convenient environments, thus eliminating the intimidation effects related to lab settings. Furthermore, it allows multiple analyses at multiple times/days and multiple experimental conditions that more closely simulate everyday experiences [28].

In a typical architecture of a wearable health monitoring system [29], wireless EEG sensors are programmed to continuously transmit EEG signal samples to a nearby edge node or a remote cloud server. This approach is challenged by the trade-off between the energy consumption of the wireless sensor and the size of transmitted data [30,31]. Therefore, it is necessary to reduce the amount of data transmitted by the sensor to increase the sensor’s lifetime. Alvarez et al. [32] have experimentally validated the energy saving from low-complexity on-sensor EEG compression that reduces the amount of data transmitted. Yet, the inevitable distortion caused by the compression algorithm questions the clinical utility of a reconstructed signal and the preservation of relevant EEG patterns.

On the other hand, embedded machine learning (EML) [33] is a promising solution where classical and deep learning models are designed to be executed within resource-constrained wearable devices to localize signal classification. However, the computational and memory requirements of machine learning algorithms challenge their implementation on embedded microcontrollers. This study demonstrates the technical feasibility and energy efficiency of an ML-based embedded EEG analysis for autism disorder spectrum detection. The technical feasibility of the proposed approach will be proven by showing, on one hand, the capability of the proposed scheme to accurately classifying ASD subjects using segments of EEG signals, and on the other, we will show the adequacy of processing the proposed algorithm in a limited resources wearable sensor.

**Figure 2:**
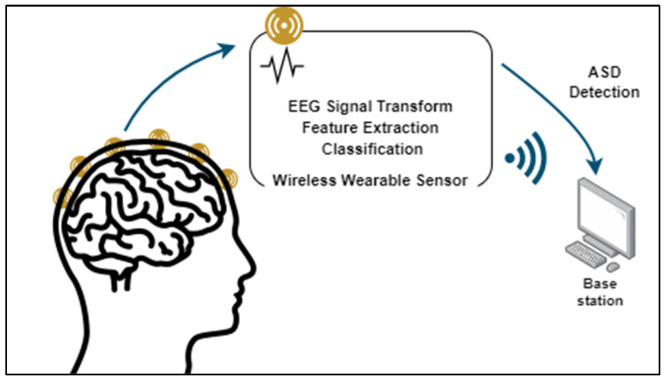
On-node EEG-based ASD detection scheme.

The main contribution is an innovative EEG-based ASD detection scheme, intended to detect ASD in early-age subjects. It performs on-node EEG signal transformation, features extraction and classification as illustrated in Figure 2.

This paper is structured as follows. We first describe the specification and the design of the ASD detection scheme as well as the processing approach of the EEG signal. We next present the performance evaluation of this scheme and its accuracy to detect ASD cases in early-age subjects. The energy efficiency is addressed in the last section of this paper to prove the adequacy of the proposed scheme for the wearable sensor. At the end of the paper, we conclude and highlight the extension of this work in the future.

## 2. Design of the ASD Detection Scheme

### 2.1. The Proposed Approach for ASD Detection

**Figure 3:**
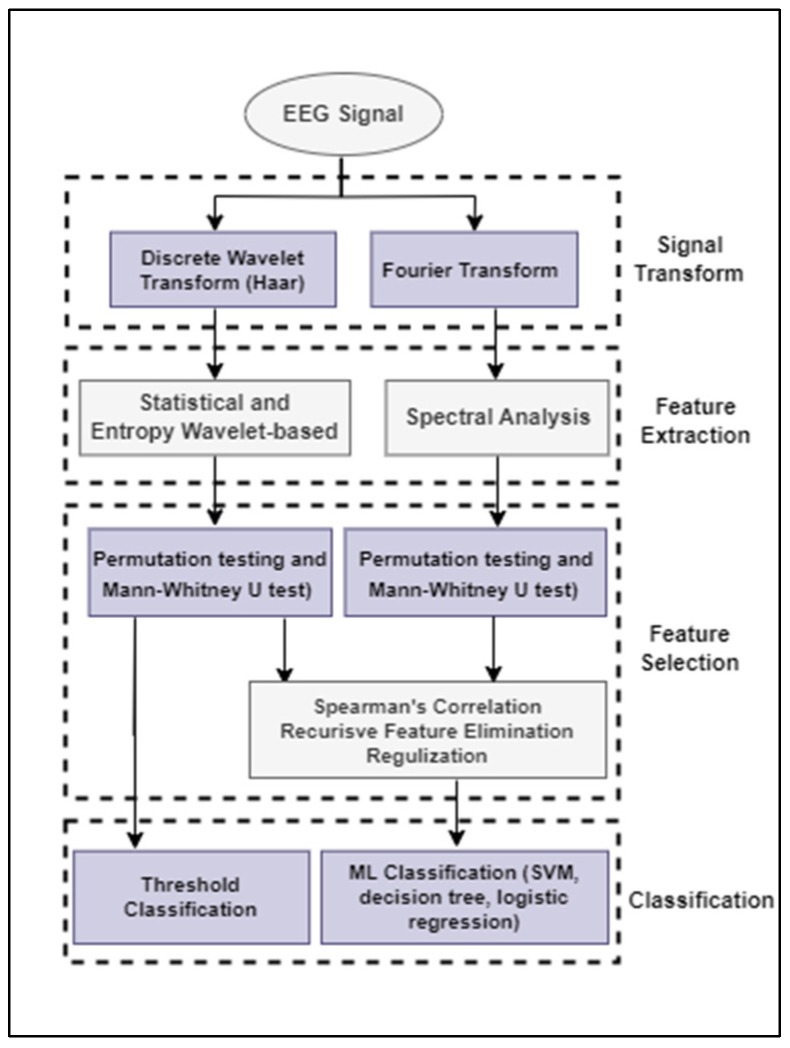
General adopted methodology for the EEG-based scheme design.

Using wearable EEG sensors can improve ASD detection as they eliminate the need for extensive wiring and allow physicians to monitor brain activity in a convenient non-intimidating environment. Figure 3 shows the modular structure of the proposed embedded EEG analysis for ASD detection. First, the EEG signal is transformed into a set of sub-signals at different frequency bands. Signal decomposition is a prerequisite for useful feature extraction that occurs in the subsequent step. The Welch’s approach of spectral analysis is applied over pre-processed overlapping windows of EEG segments. A digital wavelet decomposition is also applied to extract the wavelet statistical and information dynamics features relevant to ASD diagnosis. Based on the extracted features, an embedded classifier classifies the EEG segments. The remote backend will be notified if an ASD case has been detected. The proposed scheme requires low-complexity tasks to be processed at the sensor level due to limited resources. The classifiers that we are studying in this paper include a simple threshold classifier and embedded machine learning models, including support vector machine (SVM), logistic regression, and decision tree.

### 2.2. Signal Analysis and Feature Extraction

#### 2.2.1. Signal Transform

For efficient and relevant features extraction, the EEG signal is often decomposed using a transform method to select the appropriate frequency sub-bands. EEG signals are decomposed into a set of sub-signals at the following frequency bands: delta sub-band (0–4 Hz), theta sub-band (4–8 Hz), alpha sub-band (8–12 Hz), beta sub-band (12–30 Hz), and gamma (30–100 Hz) [34]. Each frequency band has a biological significance and reflects a distinct distribution of rhythmic activity throughout the scalp [35]. We have applied two signal transformations: wavelet transform and Fourier transform.

Wavelet transforms [36,37,38] have the ability to compress time-varying EEG signals into a small number of parameters using variable-size sliding windows that localize the EEG signal in both frequency and time domains. Discrete wavelet transform (DWT) [10,39] employs discrete scaling parameters (dilation and translation) to one single function called a mother wavelet that acts as a reference in decomposing the original signal. The dilation parameter indicates the frequency and length of the wavelet, while the translation parameter represents the shifting position. Haar wavelets [40] are the simplest and have the lowest computational complexity, making them suitable for on-sensor implementation [41].

**Figure 4:**
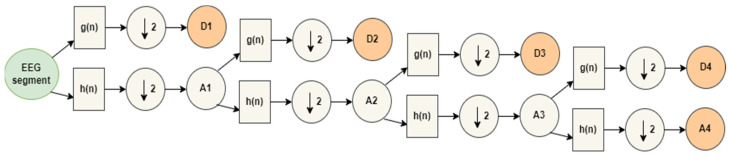
An example of a four-level decomposition of DWT.

Every level of decomposition consists of two digital filters, g(n) and h(n), and two downsamplers, as shown in Figure 4. The high pass filters, g(n), produce high-frequency components, while low pass filters, h(n), produce low-frequency components. The output of each level of decomposition is a set of details (D) and approximate (A) coefficients. Low-frequency components can be decomposed recursively according to the desired number of decomposition levels [36,42]. The number of levels for wavelet decomposition should be chosen so that the resulting frequencies closely resemble those of typical EEG sub-bands.

A four-level decomposition was used to decompose the EEG signals into detailed coefficients (D1–D4) and approximation coefficients A4. Table 1 summarizes the correspondence between wavelet coefficients and EEG frequency bands.

The Fourier transform [43,44], a mathematical procedure that decomposes any waveform into a sum of sine waves with varying frequencies, amplitudes, and phases, provides the foundation for EEG spectral analysis. It transforms the signal from the time domain into the frequency domain, allowing the analysis of the power spectrum at different frequencies.

#### 2.2.2. Feature Extraction

Different domains such as time, frequency, time-frequency, and nonlinear domains can be used for signal transformation and features extraction [45]. Compared to other techniques, statistical feature extraction and entropy-based techniques yield higher classification accuracies and are, therefore, more prevalent in EEG-based ASD detection [8]. In addition, spectral analysis is one of the dominant features proposed in the literature [5,22,46,47,48,49,50].

For the EEG spectral density, the EEG signal is not stationary over extended periods [43], which challenges the accuracy of power spectrum analysis. An improved power spectral density estimator, the Welch method [35], has been widely used in literature for EEG analysis. It involves averaging the spectral power collected over short window segments, allowing for a significant reduction in power variance.

The Welch’s approach is applied over 100 ms EEG segments with 50% overlapping after applying the Hanning window [51]. The power spectrum was computed as two values for gamma, beta, alpha, theta, and delta sub-bands: the absolute power (i.e., power in a specific frequency band) and relative power (i.e., the ratio of frequency band power to the total power over all frequency bands).

Wavelet statistical features [8,52], which represent the distribution of wavelet coefficients, are often used in EEG-based diagnosis. The wavelet statistical features used in the proposed scheme are root mean square (RMS), variance, and coefficient of variation (CV). The following Equations (1)–(3) represent the selected statistical features.

(1) $$RMS=\sqrt{\frac{\sum_{i=1}^{N}x_{i}{}^{2}}{N}}$$

(2) $$variance=\frac{1}{N-1}\overset{N}{\underset{i=1}{\sum }}{\left|x_{i}-\mu \right|}^{2},\;\mu =\frac{\sum_{i=1}^{N}x_{i}}{N}$$

(3) $$CV=\frac{\sqrt{variance}}{\mu }$$

The use of nonlinear signal processing techniques to quantify the temporal dynamics of brain activity is a novel approach [53]. Previous research has shown the significance of combining time-frequency analysis and nonlinear dynamic features for ASD detection [10]. A nonlinear feature such as entropy, a measure of uncertainty or irregularity of a system [8,53], can be used to indicate functional changes or irregularities in the brain system [53].

**Figure 5:**
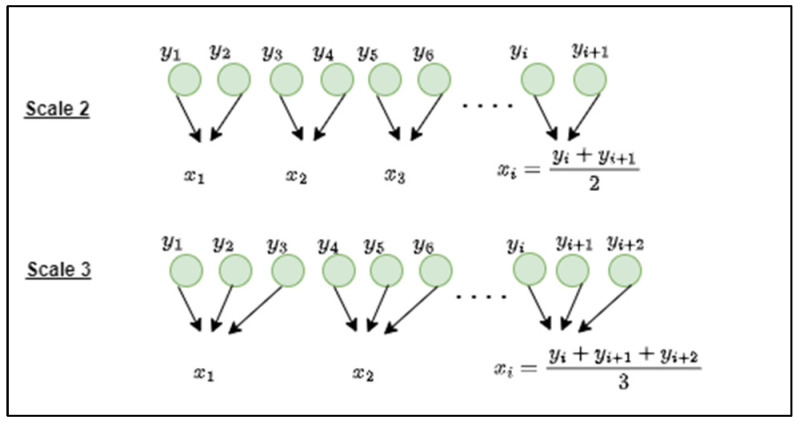
Illustration of the coarse-grained procedure.

In this paper, we adapted the multiscale entropy (MSE) technique introduced by Costa et al. in [53] to measure the complexity of brain functions at multiple time scales. Digital Haar wavelet decomposition is mathematically identical to multiple time scales coarse-graining approach for computing multiscale entropy [7] at a scale of the power of 2, as illustrated in Figure 5.

For a given time-series samples Y = (*y_1_*, *y_2_*, …., *y_n_*), the consecutive coarse-grained procedure is performed to have $\left\{x^{\tau }\right\}$, at scale vector τ using (4):

(4) $$\;x_{j}^{\tau }=\frac{1}{\tau }\;\overset{j\tau }{\underset{i=\left(j-1\right)\tau +1}{\sum }}y_{i},\;\;\;1\leq j\leq \frac{n}{\tau }$$

We applied four types of multiscale entropies: Shannon, approximate, sample, and modified sample entropies. Multiscale approximate, sample, and modified sample entropies were applied to two versions: raw features and normalized features. We have applied min-max normalization (5) that guarantees all features with the same scale.

(5) $$normalize\left(feature_{i}\right)=\frac{feature_{i}-min\left(feature\right)}{max\left(feature\right)-min\left(feature\right)}$$

Approximate entropy (ApEn) [45] represents a statistical measure of a signal’s regularity and variability over time. ApEn finds the fluctuation by comparing the signal with its delayed version [10]. Given m and r, with r being the tolerance value and m being the length of consecutive data points, u_m_(i) = (x_1 + i_, x_2 + i_, …., x_m + i_), approximate entropy is the probability of finding the similarity of a sequence with length m with the sequence of length (*m* + 1) [54], as in (6).

(6) $$ApEn\left(m,r,N\right)=\frac{1}{N-m}\;\overset{N-m}{\underset{i=1}{\sum }}\frac{\ln n_{i}^{m}}{\ln n_{i}^{m+1}}$$

$n_{i}^{m}$ stands for the number of vectors that satisfy the Euclidian distance *d_ij_^m^* between u_m_(i) and u_m_(j), less than or equal to the threshold r, as expressed in Equation (7).

(7) $$d_{ij}^{m}=\max\left\{\left|x_{i+k}-x_{j+k}\right|,\;0\leq k\leq m-1,1\leq j\leq N-m\right\}$$

We used the default values of m and r [54], m = 2, r = 0.15*standard deviation of $x^{\tau }$.

Sample Entropy (SamEn) is obtained from approximation entropy, as shown in Equation (8). It is suitable for short data sequences with low noise [45].

(8) $$SamEn\left(m,r,N\right)=\ln\frac{\sum_{i=1}^{N-m}n_{i}^{m}}{\sum_{i=1}^{N-m}n_{i}^{m+1}}$$

In sample entropy, the similarities $d_{ij}$ are computed as 0 or 1, which leads to a strict cut-off in computing similarities. The modified version of sample entropy (mSamEn) [55] computes the similarity of two segments of time series using a sigmoidal function Equation (9). The sigmoid function is the continuous and smoothed version of the 0/1 similarity function in the sample entropy.

(9) $$D_{ij}^{m}=\frac{1}{1+\exp\left[\frac{d_{ij}^{m}-0.5}{r}\right]}$$

Shannon entropy quantifies the average degree of signal uncertainty [38]. Shannon’s entropy is calculated by Equation (10).

(10) $$Shannon\;Entropy=\overset{k}{\underset{i=1}{\sum }}p\left(x_{i}\right)\log p\left(x_{i}\right)$$

where *k* is the number of unique values of *X* and *p* is the probability of these values.

For each EEG segment, 12 features are extracted to capture the irregularity of EEG and distinguish between ASD subjects and typically developing subjects.

### 2.3. Feature Selection

In our experiment, we have ten channels and 12 features, which yields 10 channels × 12 features = 120 features for each frequency band and 120 features × 5 frequency sub-bands = 600 features for each subject. Therefore, we need to minimize the feature dimension by applying the feature selection process [8,45].

To select the most significant features relevant for classification, two non-parametric statistical tests were used: Permutation testing [56] and the Mann–Whitney U-test [57], with a two-tail 95% significant interval (*p*-value < 0.05). In this approach, the feature will be considered for classification if it is significant in both statistical tests. The 600 features are, thus, reduced to 203 features for each subject.

Further reduction of features was achieved by applying supervised feature selection: filter, wrapper, and embedded methods [58,59,60]. Table 2 highlights the significant reduction in the number of features for each sub-band.

As a filter model, we have applied a non-parametric spearman correlation [57] for each frequency sub-band. A feature is removed if the correlation coefficient between two features is greater than or equal to 0.8. We have applied the recursive feature elimination (RFE) algorithm as a wrapper method. This method performs model training on a set of gradually smaller features. Every time the feature importance’s or coefficients are calculated, the lowest-scoring features are eliminated. As this method trains a model repeatedly, we must instantiate an estimator. We have used four estimators: logistic regression, perceptron, decision tree, and support vector machine. We applied recursive feature elimination (RFE) for each possible number of features. As an embedded approach, we have used regularization with goal functions that reduce fitting errors while forcing coefficients to be either small or zero.

### 2.4. Classification and ASD Detection

In computational biology, machine learning (ML) technologies have brought about a new paradigm shift [61,62,63]. Evidently, the ML-driven approach applied to clinical diagnosis has the potential to supplement traditional methods based on symptoms and external observations, intending to advance the individualized treatment plan [64]. ML approaches are fast expanding fields with applications in computational neuroscience as a result of improved neural data analysis efficiency and decoding brain function [17,61,64,65,66,67,68]. In neuroscience, the issue substantially restricts the extent and depth to which neural signatures can be functionally associated with human behaviour. These deficiencies can be addressed and solved with ML techniques [69].

In the context of ASD detection, ML algorithms demonstrated reliable and robust detection accuracy [70]. As proven by Liao et al. [68], several studies indicated that machine learning is more efficient and objective than conventional ASD diagnostic scales. In this paper, two classifiers are proposed: a simple threshold classifier and an ML classifier.

The threshold classifier is based on statistical and entropy wavelet-based features. Each feature in the test set is compared against thresholds learned from the training set. Thresholds are the mean, minimum, maximum, median, and mode of features from the ASD training set. Each feature in the testing set is classified as ASD or typically developing subjects when it crosses the threshold.

Conventional ML classification algorithms construct classification models with great precision [68]. The supervised learning model identifies the patterns and predicts the class of input data based on prior knowledge. The classification of each test data is determined by combining the features and identifying patterns in the training data. Classification consists of two stages: (1) A classification method is used for the training dataset. (2) The model generated from the training dataset is verified against a test dataset to assess the model’s performance and accuracy [59].

We have used three supervised ML classifiers: support vector machine (SVM), logistic regression, and decision tree. These classifiers have been selected due to their simplicity, high interpretability [71,72], and demonstrated accuracy in EEG-based ASD detection [7,21,22,73]. The ML classifier used a combination of spectral analysis features and statistical and entropy wavelet features.

We have adopted hyperparameter tuning to find the best model architecture. This involved creating a model for each possible combination of the specified hyperparameter values, evaluating each model and choosing the architecture that yields the best results. Table 3 shows the tuned hyperparameters and their values for each classifier.

To implement the ML classifier in the sensor, we extracted the SVM and logistic regression weights/coefficients offline to form the decision classification Equations (11) and (12). For the decision tree, we trained the model offline. Then, the resulting if-then rules were included in the sensor.

#### 2.4.1. Support Vector Machine

Support vector machine (SVM) is a classifier that separates the two data classes using a hyperplane. The set of data points with the shortest distance from the hyperplane is known as the support vector. Using support vectors, the hyperplane is positioned to maximize the margin, which is a metric that indicates the distance between two classes [74,75].

The SVM technique has been selected due to its high performance with small data sets [76]. We have used a linear kernel, as shown in Equation (11). In Python’s scikit-learn module [77], the weights/coefficients are assigned to the features and can be extracted only if the kernel is linear.

(11) $$w^{T}\;x\;+\;b\;=0$$

where *w^T^* is the weight/coefficient vector for the feature vector *x*, and *b* is the bias [78].

#### 2.4.2. Logistic Regression

In medicine and biology, binary classification problems are frequently solved using logistic regression [71]. Logistic regression describes the link between one dependent binary variable and one or more independent variables using a logistic function to predict the probability of a categorical outcome [13,61]. The logistic regression process is shown in Equation (12).

(12) $$Sigmoid\left(z\right)=\frac{1}{1+e^{-z}},\;\;\;z=w\;x\;+\;b$$

where *w* is the weight/coefficient vector for the feature vector *x*, and *b* is the bias [13].

#### 2.4.3. Decision Tree

Decision tree is among the most used classifiers in machine learning [79]. The decision tree can reduce complex decision processes into a succession of simpler decisions. The decision tree is a tree that is governed by if-then rules. The tree nodes are questions about the features, representing each answer as a child node. The tree leaves are the classification label [74,78]. The dataset is repeatedly subdivided for binary classification. Optimal partitioning points must be chosen during this procedure [79]. A criterion minimizes the probability of misclassification, including entropy and Gini index, as shown in Equations (13) and (14), where *p_j_* is the probability of classifying.

(13) $$Entropy=-\underset{j}{\sum }p_{j}\log_{2}p_{j}$$

(14) $$Gini=1-\underset{j}{\sum }p_{j}^{2}$$

## 3. Performances Evaluation of the Proposed Scheme

### 3.1. Classification of ASD Cases

For classification, we used EEG signals provided from a publicly accessible dataset from Catarino et al. [80] study. This dataset includes 15 ASD subjects (mean = 31 months, standard deviation = 6) and 15 typically developing subjects (mean = 29 months, standard deviation = 4). All subjects were right-handed males. Clinical psychologists diagnosed individuals with ASD using worldwide diagnostic criteria. Post-visual stimuli data were obtained using a 32-channel system corresponding to the international 10-20 system [81] and a reference electrode at the tip of the nose. The data were sampled with a bandpass filter between 0.1 and 50 Hz at a sampling rate of 1000 Hz. The number of epochs included in the study [80] was for ASD patients (mean = 81, SD = 8) and for typically developing subjects (mean = 83, SD = 7). The length of the signal is 400 ms post-stimulus period. Ten channels were included in the [80] dataset: P8, TP8, T8, P7, FT8, TP7, F8, T7, FT8, and F7.

For the performance evaluation, the proposed scheme was implemented using MATLAB and Python. A cross-validation approach was adopted in our case because of the small number of subjects. We used the k-fold cross-validation method (k = 5), where the dataset is randomly divided into k (k = 5) partitions of equal size, with one partition used for testing and the rest for training for each of the k iterations [8]. The classification performance of the ASD subject based on the extracted features is determined by averaging the five-fold performance findings. We were interested in evaluating the scheme’s accuracy [21], sensitivity [5,7], specificity [5,7], positive predictive value [5,7], negative predictive value [5], and F1-score [79]. The mathematical equations for the performance metrics are given by the following Equations (15)–(20):

- Accuracy (*Acc*): It provides the correct prediction of the classifier.

(15) $$Acc=\frac{TN+TP}{total}$$

- Sensitivity or recall (*Sen*): It expresses the ability of the scheme to identify subjects who have ASD correctly.

(16) $$Sen=\frac{TP}{TP+FN}$$

- Specificity (*Spec*): It shows the scheme’s ability to identify typical developing subjects correctly.

(17) $$Spec=\frac{TN}{TN+FP}$$

- Positive Predictive Value (*PPV*) or Precision: It provides the probability of how likely it is that the subject has ASD.

(18) $$PPV=\frac{TP}{TP+FP}$$

- Negative Predictive Value (*NPV*): It expresses the probability of how likely it is that the subject is a typical developing subject.

(19) $$NPV=\frac{TN}{TN+FN}$$

- F1-score (*F*1): It combines both sensitivity and PPV in a single metric.

(20) $$F1=\frac{2*Sen*PPV}{Sen+PPV}$$

where *TP*, *FP*, *TN*, *FN*, respectively, represent true positive, false positive, true negative, and false negative.

As explained in the previous section, we studied two scenarios to evaluate the capability of the proposed scheme for ASD recognition. The first scenario is based on the idea of using wavelet-based features with the application of the threshold classification approach. In the second approach, we extended the features to have the spectral analysis features, and the classification was performed based on ML classifiers.

Table 4 summarises the classification results for the first studied scenario based on the threshold classifier [82]. This table shows that the classification based on multiscale approximation entropy was capable of achieving high accuracy in only the beta and alpha sub-bands. The highest accuracy of 86% was obtained in the alpha sub-band using channel P7.

To improve the accuracy in the alpha sub-band, we combined this feature with other features that have 100% NPV or PPV. We used a set of if-then-else rules for the classification decision that allows first to consider the feature that provided 100% NPV or PPV; then, the Multiscale approximation entropy in the alpha-band is applied. We noted that the accuracy was enhanced to 93% when we combined the multiscale approximate entropy in the alpha sub-band in channel P7 with multiscale approximate entropy in channel P8 in the gamma sub-band. However, we still have low sensitivity (86.6%), as shown in Table 4.

**Figure 6:**
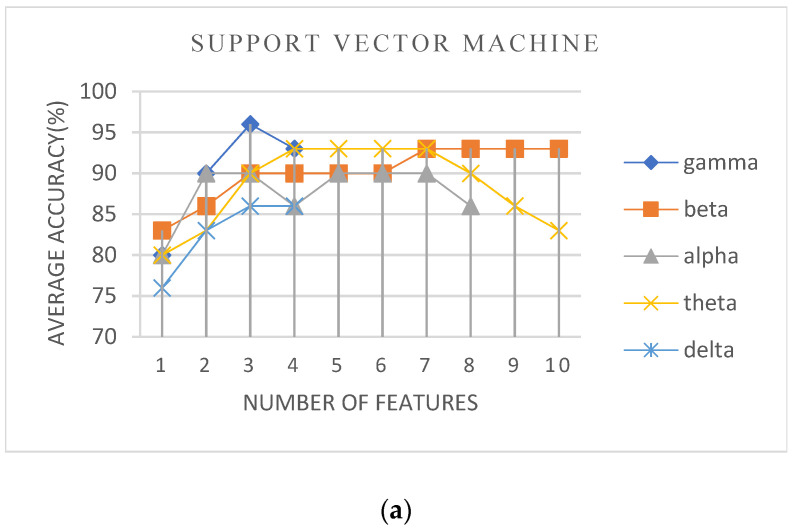

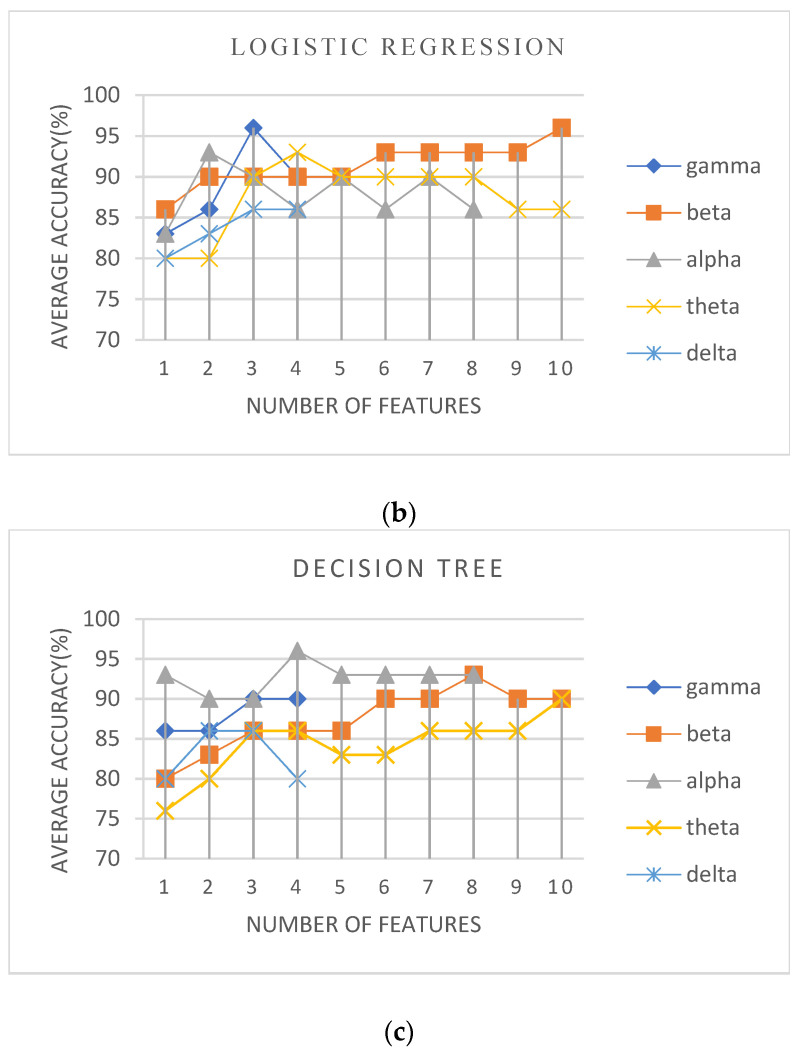
Averaged accuracy variations with the number of features selected by RFE for the ML classifiers for each sub-band. (**a**) support vector machine, (**b**) logistic regression, (**c**) decision tree.

In the second scenario, the training experiments were conducted using K80, T4, and P100 GPU with 52 GB RAM and 8 cores Intel(R) Xeon(R) CPU @ 2.2 GHz. We studied the classification accuracy of the different EEG subjects in the data sets using each possible combination of features resulting from the Recursive Feature Elimination (RFE) algorithm for all combinations of machine learning (ML) hyperparameters values. Figure 6 presents the best overall accuracy values obtained with the classification based on the deployment of different ML algorithms with hyperparameters tuning. From this figure, we can note that the classification with all ML algorithms achieved an accuracy score of 96% in classifying ASD cases. This accuracy is higher than the performance achieved with the threshold classifier approach. This reflects the capability of employing ML models to recognize a pattern that could achieve reliable and accurate diagnostics.

In Figure 6a,b, we can see that the use of SVM and logistic regression algorithms achieved the highest accuracy score with three selected features in the gamma sub-band: absolute Welch, multiscale approximate entropy, and normalized multiscale approximate entropy. The logistic regression algorithm has also achieved an accuracy of 96%, using ten features in the beta sub-band. For a wearable body sensor, adopting fewer features is adequate for low-processing capabilities. With the use of the decision tree classifier, Figure 6c shows that the highest accuracy, 96%, was obtained with the adoption of four selected features in the alpha sub-band: absolute Welch, relative Welch, normalized multiscale approximate entropy, and variance. The results validate the role of feature selection in choosing a subset of highly discriminating features capable of distinguishing samples from distinct classes. Moreover, results prove that too many irrelevant or redundant features in the data can reduce the accuracy of the ML models [59,60].

Based on the previous discussion of the results, the best classification accuracies were obtained in the gamma and alpha sub-bands. Gamma frequency oscillations have been linked to various brain activities, such as attention and visual perception, including object perception [83]. On the other hand, Orekohova et al. demonstrated that alpha rhythm is associated with attention activities such as visual stimuli [18,19,82]. Additionally, the alpha rhythm is less susceptible to muscle and movement artifacts. Moreover, individual differences in emotional and cognitive involvement have a reduced effect on alpha activity. We, therefore, anticipated that inter-individual differences in these uncontrolled parameters during passive stimulus viewing would contribute less to the alpha sub-band [19].

The best selection set of hyperparameters applied to ML algorithms and features used for the accurate classification results are presented in Table 5. From this table, we can observe that multiscale approximate entropy and spectral power (Welch) are the best features for accurate classification to achieve the best discrimination. In depth, approximate entropy is efficient when deployed to calculate the complexity of time-series data, even in the presence of artifacts [84]. It is also suitable for short data, as in our case [85]. The spectral power (Welch) improves the precision of traditional spectral analysis. Because of the EEG nonstationary propriety, Welch’s approach, which involves averaging the spectral power collected over short window segments, reduces this variance significantly.

Despite major differences in how machine learning algorithms are operating, we note from Table 6 that they were all capable of classifying the different EEG signals with high accuracy, sensitivity, and F1 scores. Compared to the threshold classifier, we can say that both the accuracy and sensitivity metrics were significantly enhanced. In depth, the accuracy is increased from 93% to 96%, while the sensitivity has been elevated from 86% to 100%. This result attests the technical feasibility of the proposed approach for efficient detection of ASD cases with the deployment of the described processing techniques.

Compared to similar EEG-based ASD detection studies, we can note that our proposed scheme outperforms all stated similar schemes reported in the literature, as presented in Table 6. To conduct this comparison, we implemented the feature extraction and classification phases of [5,7,21] with the same dataset that we used. Furthermore, we evaluated the same adopted performance metrics with five-fold cross-validation.

Bosl et al. in [7] used the Daubechies (DB4) wavelet for multiscale decomposition. They extracted nonlinear features from each frequency band: recurrence quantitative analysis, detrended fluctuation analysis, and sample entropy. They used the SVM algorithm for classification with default values of hyperparameters.

Gabard-Durnam et al. [5] used the power spectral of EEG signal with logistic regression classifier. Zhao et al. [21] employed singular spectrum analysis (SSA) to extract the desired alpha rhythm and fed individual alpha peak frequency and individual alpha absolute power features into linear SVM. Bosl et al. [7] achieved a classification accuracy up to 63%, while [5,21] had 73%. The F1 score was in the range of 64–72%. The low performance of these studies may relate to the variation of studies subjects’ ages, experiment designs, extracted features, and/or classifiers. 

The highest detection performance of our EEG-based ASD detection scheme clearly indicates that early ASD biomarkers can be extracted from EEG. Time-frequency EEG decomposition, nonlinear features, and spectral power (Welch approach) are promising automated assistive tools for ASD detection that can reduce the bias of the behavioural-based EEG diagnosis and optimize the time and effort of neurologists.

### 3.2. Energy-Consumption Estimation of the Proposed Scheme

The energy consumption of the proposed scheme was performed using Contiki-NG [86,87], an open-source Internet of Things operating system. It is intended for low-power microcontroller emulation. It is integrated with Cooja that allows the emulation of some motes such as Zolertia Z1 platform, which was adopted in our study. The sensor Z1 platform is based on a low-power MSP430 microcontroller with IEEE 802.15.4 radio modules [87].

Contiki-NG uses the Energest module that is capable of estimating the energy and the time related to the processing of a given task. It also estimates the energy related to the radio activities for the transmission and reception of data. Using this information along with the hardware power consumption model according to the mote datasheet, the developer can estimate the system’s energy usage.

The energy for each Energest state is expressed by Equations (21)–(23). We have computed energy consumption for the CPU state, the radio transmitting state, and the whole system. The whole system energy consumption is evaluated by summing the values of all tracked states.

(21) $$Current_{state}\left(mA\right)=\frac{{\mathrm{ticks}}_{\mathrm{state}}\;*\;\mathrm{current}\_HW_{\mathrm{state}}}{RTIMER\_ARCH\_SECOND\;*\;Execution\_time_{sec}}$$

(22) $$Power_{state}\;\left(mW\right)=Current_{state}*voltage$$

(23) $${\mathrm{Energy}}_{state}\;\left(\;mJ\right)=Power_{state}*\;Execution\_time_{sec}$$

where $ticks_{state}$ is the number of clock cycles a system has spent in a state obtained from the Energest module. The $\mathrm{current}\_HW_{\mathrm{state}}$ is the current state provided from the mote datasheet. RTIMER_ARCH_SECOND is a mote-specific number of ticks per second.

We assumed that the data segment is already acquired since the data acquisition has the same energy consumption for all scenarios. We studied the following scenarios for the energy evaluation:

- **On-node feature extraction and classification**: In this scenario, we evaluated the energy consumption related to the processing of the EEG signal and the extraction of the features and the classification at the wearable sensor. We implemented the process related to the extraction of the features that provided the highest accuracy, 96%, in our scheme (Table 6).
  ∘ For the classification with SVM and logistic regression, the EEG signal was processed in the gamma sub-band. We evaluated the deployment of the best performance features (absolute Welch, ApEn, and ApEn normalized). For the classification, we added the decision classification Equation (11) for SVM and Equation (12) for logistic regression.
  ∘ For the classification with the decision tree algorithm, the EEG signal was processed in the alpha sub-band. The energy consumption was evaluated for four features of the proposed scheme (absolute Welch, relative Welch, variance, and ApEn normalized). For the classification, we have added the if-else rules resulting from the decision tree model.
- **Streaming raw EEG signal segment**: This scenario is based on the idea of streaming raw EEG signal as in the traditional computerized scheme.

Table 7 shows the results of the execution time in CPU Energest module time results for each different feature proposed to be used for classification in the designed scheme. We can see that the same extracted wavelet features with the classification with the decision tree model generally require less processing time and consequently less energy consumption. In depth, with the application of the decision tree, the features are extracted in the alpha sub-band with fewer wavelet coefficients than the gamma sub-band in SVM or logistic regression classifiers because of the down-sampling process. For example, ApEn normalized feature in the gamma sub-band consumes 217,575 clock cycles at the CPU processing while it takes only 14,957 clock cycles in the alpha sub-band.

For the spectral analysis feature, the computational energy consumption is higher than the energy consumption of the extraction of the other features. The lowest computation energy consumption is in the variance feature.

**Figure 7:**
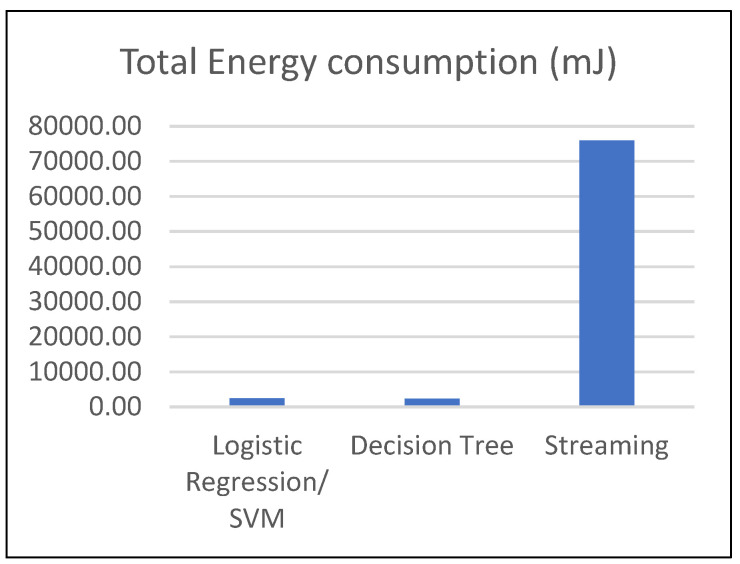
Total energy consumption results in our scheme compared with the streaming scenario.

Table 8 shows each ML model’s energy consumption (transmit, CPU, total energy) in our scheme and streaming scenario. Figure 7 shows a comparison of the energy consumption between the two scenarios. We can see that streaming the whole EEG signal for classification in a remote server needs high energy compared to detecting the ASD disorder with the execution of the proposed scheme in the wearable sensor. A gain of around 97% of energy is ensured while executing the proposed scheme with the decision tree algorithm. This result attests to the proposed scheme’s energy efficiency and its adequacy for on-node processing.

From another side, we can also see that the decision tree classifier based on different features requires less energy consumption than the deployment of an SVM classifier or logistic regression algorithm. This difference in energy consumption is mainly related to the fact that the decision tree is extracting the features in the alpha sub-band, which requires less computation than the gamma sub-band, during signal transformation.

The different tasks implemented in the proposed scheme were selected to meet the requirement of low complexity for the adequacy of embedded processing. The results presented in Figure 7 and in Table 7 and Table 8 demonstrate that the scheme can be efficiently processed in a wearable sensor with limited capabilities attesting about the feasibility of this solution.

## 4. Conclusions

This paper advocates EEG signals as an objective diagnostic tool for ASD detection in early-age subjects. It demonstrates the technical feasibility of this approach by showing the adequacy of the proposed scheme to be processed in a wearable sensor with limited processing capabilities while maintaining an accurate level of detecting ASD cases. It also attests the energy efficiency of the proposed ML-based embedded EEG analysis for ASD detection with a high level of energy saving. Results have shown that the on-node feature extraction and classification scheme strike the balance of energy efficiency and high accuracy using a combination of nonlinear analysis, multiscale approximate entropies in the time-frequency domain, and spectral analysis (Welch) of EEG signals. The embedded implementation of SVM, logistic regression, and decision trees has reached an accuracy of 96% and has proven to be more energy efficient than typical streaming of non-processed EEG samples. The decision tree yields the highest energy savings, around 97%.

As to future works, we are interested in in prototyping the proposed scheme as wearable wireless sensor for in-laboratory experimental deployment, which will help study the effect of classification under data collected by untrained people in uncontrolled environments. In addition, we also think that using larger datasets with an adequate Convolutional Neural Network architecture might contribute to design a scalable and highly accurate assistive tool in clinical decisions. While the use of deep neural networks might be a powerful tool for efficient classification [88], the feasibility of this idea requires performing optimization techniques that can significantly compress the overall classification model size [33,89]. Further optimizations are also required, such as a runtime optimization of the model [90].

## Figures and Tables

**Table 1:** Frequency sub-bands for each wavelet coefficient.

Wavelet Coefficient and Its Frequency	EEG Approximate Label
D1 (25 Hz–50 Hz)	Gamma
D2 (12 Hz–25 Hz)	Beta
D3 (6 Hz–12 Hz)	Alpha
D4 (3 Hz–6 Hz)	Theta
A4 (0.1 Hz–3 Hz)	Delta

**Table 2:** The number of features after applying feature selection methods.

Frequency Sub-Band	Number of Features	Number of Features after Permutation and Mann Whitney	Number of Features after Spearman Correlation
gamma	120	25	4
beta	120	56	10
alpha	120	56	8
theta	120	44	10
delta	120	22	4

**Table 3:** Hyperparameters tuning.

Classifier	Hyperparameter	Values
Support Vector Machine	Kernel	linear
	regularization parameter, C	0.01, 0.1, 1, 10, 100
Logistic Regression	Solver	liblinear, newton-cg, lbfgs
	regularization parameter, C	0.01, 0.1, 1, 10, 100
Decision Tree	Criterion	gini, entropy
	Splitter	random, best

**Table 4:** The best performance measures of threshold classifier.

Number of Features	Sub-Band	Acc	Sen	Spec	PPV	NPV	F1
1	Beta	83.33	93.33	73.33	77.78	91.67	84.85
1	Beta	83.33	93.33	73.33	77.78	91.67	84.85
1	Alpha	83.33	80	86.67	85.71	81.25	82.76
1	Alpha	86.67	86.67	86.67	86.67	86.67	86.67
2	alpha/gamma	93.3	86.67	100	100	88.2	92.86

**Table 5:** Features resulting from RFE and model hyperparameter values for the best performances of ML algorithms.

Classifier	Sub-Band	Number of Features	Channel, Features Resulting from RFE	Hyperparameter	Hyperparameter Value
SVM	Gamma	3	TP7, absolute Welch	Kernel C	Linear 10
			P8, ApEn		
			F7, ApEn Normalized		
Logistic Regression	Gamma	3	TP7, absolute Welch	Solver C	liblinear, newton-cg, lbfgs 10, 100
			P8, ApEn		
			F7, ApEn Normalized		
Decision Tree	Alpha	4	P7, Variance	criterion splitter	entropy random
			T8, absolute Welch		
			TP7, relative Welch		
			TP8, ApEn Normalized		

**Table 6:** The performance of our scheme compared with the benchmarks.

Study	Classifier	Acc	Sen	Spec	PPV	NPV	F1
Our Scheme	Threshold	93.3	86.67	100	100	88.2	92.86
	SVM	96.67	100	95	93.33	100	96.55
	Logistic Regression	96.67	100	95	93.33	100	96.55
	Decision Tree	96.67	100	96	90	100	94.74
Bosl et al. [7]	SVM	63.33	95	35	59	90	72.79
Gabard-Durnam et al. [22]	Logistic Regression	73.33	73.33	75	58.33	80	64.98
Zhao et al. [21]	SVM	73.33	79.33	56.67	67.67	68.33	73.04

**Table 7:** The Energest module time results of each feature in our scheme.

ML Classification Algorithm	Logistic Regression/SVM			Decision Tree			
**Sub-band, Extracted Features**	gamma, absolute Welch	gamma, ApEn	gamma, ApEn Normalized	alpha, variance	alpha, absolute Welch	alpha, relative Welch	alpha, ApEn Normalized
**CPU (ticks)** ^1^	322,124	217,377	217,575	851	322,096	322,108	14,957
**Total Time (ticks)** ^2^	387,115	294,830	294,830	98,222	387,115	387,115	98,222

^1^ The number of clock cycles a system has spent in CPU state. ^2^ The number of clock cycles a system has spent in all states.

**Table 8:** The energy consumption of our proposed scheme and streaming scenario.

Scheme	On-Node Feature Extraction and Classification		Streaming
	Logistic Regression/SVM	Decision Tree	
**Total Time (ticks)** ^1^	976,775	970,674	43,579,790
**CPU (ticks)** ^2^	757,076	660,012	1,062,120
**Radio Tx (ticks)** ^3^	102	102	189,570
**Radio Rx (ticks)** ^4^	976,673	970,572	43,390,220
**Transmit Energy consumption (mJ)** ^5^	0.16	0.16	301.99
**CPU Energy consumption (mJ)** ^6^	693.12	604.26	972.40
**Total Energy consumption (mJ)** ^7^	2374.33	2274.96	75,957.26

^1^ The number of clock cycles a system has spent in all states. ^2^ The number of clock cycles a system has spent in CPU state. ^3^ The number of clock cycles a system has spent in radio transmitting state. ^4^ The number of clock cycles a system has spent in radio receiving state. ^5^ The system energy consumption evaluated for radio transmitting state. ^6^ The system energy consumption evaluated for CPU state. ^7^ The summed system energy consumption evaluated for all tracked states.
